# Nucleolar responses to DNA double-strand breaks

**DOI:** 10.1093/nar/gkv1312

**Published:** 2015-11-28

**Authors:** Dorthe Helena Larsen, Manuel Stucki

**Affiliations:** 1 Danish Cancer Society Research Center, Strandboulevarden 49, 2100 Copenhagen, Denmark; 2 Department of Gynecology, University of Zurich, Wagistrasse 14, CH-8952 Schlieren, Switzerland

## Abstract

Maintenance of cellular homeostasis is key to prevent transformation and disease. The cellular response to DNA double-strand breaks, primarily orchestrated by the ATM/ATR kinases is one of many mechanisms that serve to uphold genome stability and homeostasis. Upon detection of double-strand breaks (DSBs), several signaling cascades are activated to halt cell cycle progression and initiate repair. Furthermore, the DNA damage response (DDR) controls cellular processes such as transcription, splicing and metabolism. Recent studies have uncovered aspects of how the DDR operates within nucleoli. It appears that the DDR controls transcription in the nucleoli, not only when DNA breaks occur in the rDNA repeats, but also when a nuclear DDR is activated. In addition, we have gained first insights into how repair of DSBs is organized in the nucleolus. Collectively, these recent studies provide a more comprehensive picture of how the DDR regulates basic cellular functions to maintain cellular homeostasis. In this review we will summarize recent findings and discuss their implications for our understanding of how the DDR regulates transcription and repair in the nucleolus.

## INTRODUCTION

### The DNA damage response

The maintenance of genomic integrity is crucial for cell survival and for preventing malignant transformation. In all living cells DNA is constantly exposed to damage generated both by endogenous and exogenous sources, thus threatening genomic stability. To ensure genomic integrity, mammalian cells have evolved a sophisticated and complex network of pathways, collectively termed the DNA damage response (DDR), which inspects the genome for the presence of damage. The DDR network detects and signals aberrant DNA structures, activates DNA repair pathways and regulates a broad spectrum of fundamental cellular processes. Such processes include DNA replication, cell cycle progression, apoptosis, senescence and transcription (1,2).

DNA double-strand breaks (DSBs) represent the most deleterious form of DNA lesions. DSBs can arise spontaneously during DNA replication when arrested replication forks collapse. In addition, DSBs can form in response to certain exogenous clastogens such as ionizing radiation (IR) or radiomimetic drugs. In some cases DSBs are formed as a part of a normal physiological program, for example during V(D)J and class switch recombination in developing lymphocytes (3). If unrepaired or aberrantly repaired, DSBs may potentially give rise to chromosomal rearrangements and aneuploidy, which are the underlying cause of several human disorders, such as developmental and neurological diseases, as well as cancer (4). In mammalian cells DSBs are mainly repaired by two different repair pathways: the error prone non-homolgous end joining pathway (NHEJ) and error-free homology-directed repair (HDR). NHEJ is the major repair pathway in response to DSBs but complexity of the breaks and the local chromatin environment may cause NHEJ to fail thus promoting a switch to HDR in the S- and G2 phases of the cell cycle when a sister chromatid is present that can be used as a template for repair (5). The DSB repair pathway choice is highly regulated during the cell cycle and through DNA damage-induced signaling mechanisms (3).

The question if the cellular response to DSBs is uniform throughout the cell nucleus or if regions exist where the response is differently regulated is the subject of ongoing research efforts. Especially the existence of DSBs in repetitive sequences such as those present at telomeres, in satellite repeats and in the ribosomal gene arrays may pose a challenge for the cell to repair because such regions are highly recombinogenic. Recent evidence suggests that the cellular response to DSBs within the nucleoli that contain the ribosomal gene arrays has unique features, which we will review in the sections below.

### Nucleolar structure and functions

The nucleolus is a membrane-less organelle found in the nucleus of eukaryotic cells formed around a distinct part of the genome: the ribosomal DNA (rDNA) genes. Each cell contains more than 200 copies of rDNA genes with each module containing a 30 kb intergenic spacer and a 14 kb precursor coding region (6). In human cells, the rDNA genes are arranged in clusters also known as Nucleolar Organizer Regions (NORs) on the five acrocentric chromosomes. Actively transcribed NORs mediate the assembly of nucleoli and consequently, nucleolar structure and transcription are closely inter-linked (7).

The rDNA repeats within the nucleolus are found in two forms: an open, highly-transcribed conformation and in a silenced heterochromatin state (8,9). While the physiological role of two such opposing chromatin states is not thoroughly understood it is clear that ribosomal RNA (rRNA) transcription can be regulated both at the level of the individual repeats as well as by the number of active repeats (6).

Nucleolar transcription is mediated by RNA Polymerase I (RNA Pol I) and a large number of co-factors in the nucleolus (9–11). The minimal requirement for initiation of rDNA transcription is the presence of the upstream binding factor (UBF), the promoter selectivity factor (SL1) and the RNA Pol I complex at the rDNA promoter. rDNA transcription generates the unstable 47S pre-rRNA transcript. This transcript is rapidly processed and post-transcriptionally modified giving rise to the 18S, 5.8S and the 28S transcripts, which are the nucleic acid building blocks required for ribosome biogenesis (12).

Additionally, an important role of the nucleolus as a sensor of cellular stresses is emerging. Pathways activated by genotoxic, metabolic, osmotic and transcriptional stress all converge on RNA Pol I and result in inhibition of rRNA transcription and release of ribosomal proteins from the nucleolus as a consequence (10). The released ribosomal proteins sequester the ubiquitin ligase Mdm2 in the nucleoplasm and thereby boost p53 levels leading to cell cycle arrest, senescence or apoptosis (13). Nucleolar stress can also induce p53-independent cell cycle arrest and senescence although the mechanism is not equally well defined (14–16).

### The DDR in the context of nucleolar chromatin

All rDNA genes share the same sequence but are packed in either a highly condensed silenced heterochromatic structure or an actively transcribed undercondensed confirmation. The interior of the nucleolus has very low concentration of DNA which can be experimentally evidenced by lack of DAPI staining. The nucleolar DDR must operate in accordance to these atypical features to ensure preservation of genome stability.

The highly repetitive nature of rDNA repeats makes them particularly sensitive to unscheduled recombination. Studies in yeast have shown that DSBs are sensed and processed within the nucleoli but homologous recombination is actively excluded from the nucleoli by Smc5 and Smc6 in a sumoylation-dependent manner (17). DSBs in rDNA are transiently relocated to extranucleolar sites for recombinational repair. Smc5 and Smc6 mutants display a rDNA hyper-recombination phenotype with progressive loss of rDNA repeats as a consequence (17). These findings highlight the vulnerability of rDNA repeats and the special requirement for regulation of homology-dependent repair in the nucleolus. Evidence of rDNA sensitivity in mammalian cells comes from Bloom syndrome patients with mutated BLM protein. Cells lacking functional BLM display increased rates of sister chromatid exchange and have highly unstable rDNA arrays with significant genomic rearrangements (18).

The active rDNA repeats are the most highly transcribed sequences in the genome with tightly packed RNA Pol I along the repeats (7). The high density of RNA Pol I in rDNA increases the risk of collision between the transcription and repair- or replication-machinery and extraordinary measures may therefore be required to prevent collision in highly transcribed regions of rDNA. To prevent collision between replication and rRNA transcription a replication fork barrier (RFB) is formed downstream of the coding region in actively transcribed rDNA. Inhibition of the RFB impedes replication fork progression in a transcription dependent manner suggesting impairment of fork movement by head-on collision with the transcription machinery (19). Furthermore, highly transcribed repeats in yeast require silent repeats for efficient assembly of repair factors (20) exemplifying additional adaptation of DSB repair in open nucleolar chromatin.

Silenced nucleolar heterochromatin presents yet another obstacle to DSB repair. The silenced rDNA repeats feature epigenetic characteristics similar to constitutive heterochromatin including CpG methylation and histone modifications such as H3K9me2/3 and H4K20me3, and are highly condensed (21). Heterochromatic domains in general present a physical barrier to DSB repair and specifically requires phosphorylation of the heterochromatin formation protein Kap-1 by the ATM kinase (22). Phosphorylated Kap-1 mediates release of the chromatin remodeling factor CHD3 allowing decondensation of chromatin in the vicinity of the lesion and thus efficient DSB repair to take place (23).

The nucleolus therefore represents a highly specialized genomic domain that is a challenge for the cell to maintain and sustain in the context of DNA damage signaling and DNA repair. Our current understanding of how the cellular response has adopted to these challenges is only beginning to emerge. Several studies have recently been published addressing how the DDR signals to and operates within, the nucleoli.

## RESPONSE TO DSBs IN NUCLEOLAR CHROMATIN

An essential piece of the puzzle to understand the nucleolar DDR came from the Casellas lab in 2007. Kruhlak and colleages observed a transient block in rRNA synthesis upon treatment of primary mouse embryonic fibroblasts (MEFs) with γ-irradiation (24). To investigate the nature of the transcriptional silencing the authors introduced DSBs locally in individual nucleoli using laser micro-irradiation. It appeared that the inhibition did not spread to surrounding nucleoli thus suggested in *cis* inhibition of RNA Pol I transcription in the proximity to the breaks. Cells irradiated with 10 Gy also displayed segregation of the nucleolar proteins UBF into nucleolar caps, a hallmark of transcriptionally inactive cells (24,25).

Kruhlak et al. further demonstrated that RNA Pol I transcription is not blocked by the damage itself but is controlled by the DNA damage kinase ATM. The inhibition could be abrogated by chemical inhibitors against ATM and ATM ^−/−^ MEFs were unable to downregulate RNA Pol I transcription in response to DNA damage (24).

To assess the importance of other DDR factors in DNA damage-dependent regulation of RNA Pol I transcription, a panel of repair-deficient knock-out MEFs were screened. This analysis revealed that after γ-irradiation the two DDR adaptor proteins NBS1 and MDC1 were required for efficient transcriptional inhibition, whereas Ku, BRCA1, 53BP1 and H2AX were dispensable (24).

Mathematical modeling of fluorescence recovery after photobleaching (FRAP) experiments suggested that the molecular mechanism underlying local DNA damage-dependent RNA Pol I inhibition involved both inhibition of initiation complex assembly as well as progressive displacement of elongating Pol I holoenzymes from DNA. The model was further supported by chromatin immunoprecipitation showing loss of RNA Pol I association with actively transcribed regions of rDNA upon induction of DSBs by γ-irradiation (24). The RNA Pol I displacement model was also supported by results obtained using mass spectrometry after IR (26).

## REPAIR OF DSBs IN NUCLEOLAR CHROMATIN

A long-standing question in the field of nucleolar biology is how DSBs are repaired in the nucleolus of mammalian cells. This issue was addressed for the first time in two recent studies by the McStay and Greenberg laboratories. The homing endonuclease I-PpoI from *Physarum polycephalum* was used to induce DSBs within the rDNA repeats. I-PpoI has a 15 base-pair recognition site in the 28S coding sequence of each of the approximately 200 rDNA repeats.

Induction of DSBs within the rDNA repeats by I-PpoI expression confirmed inhibition of nucleolar transcription upon induction of DSBs in rDNA and segregation of nucleolar structure into nucleolar caps as previously reported by Kruhlak et al. (see above). Also in the case of DSB induction by I-PpoI expression, both rRNA silencing and nucleolar cap formation were dependent on ATM signaling (27,28). ATM was furthermore shown to be present in the nucleoli prior to damage and activated ATM localized to nucleolar caps (27).

It seems that non-homologous end-joining (NHEJ) is the primary repair pathway of DSBs in rDNA (28). Chemical inhibitors against DNA-PK, transient depletion of NHEJ factors by siRNA transfection and genetically defined mouse fibroblasts (DNA-PK^−/−^) all demonstrated significantly increased numbers of DSBs and exacerbated ATM dependent silencing after I-PpoI induction. Transient depletion or conditional knock-out of homology directed repair (HDR) factors did not significantly increase the number of DSBs (28), thus suggesting that NHEJ is the predominant repair pathway for DSBs in rDNA as it is for DSBs in the rest of the genome (5).

The precise localization of DSB repair by NHEJ in rDNA is not yet clear. 53BP1 localizes to nucleolar caps and associates with DSBs but factors like ku80 and XRCC4 could not be detected within nucleolar caps (27,28). Previously, Ku70, Ku80 and the catalytic subunit of DNA-PK were detected in purified nucleoli (26) and HNEJ factors may therefore associate with the DNA lesions already within the nucleoli. Detailed temporal analysis of the repair kinetics and cap formation dynamics can provide further insight into the coordination of the two processes.

The study by van Sluis and colleagues suggests that HR is also involved in the repair of DSBs in rDNA even if this may concern a smaller fraction of the total number of DSBs after I-PpoI induction. Nucleolar caps stained positive for rDNA, the DNA damage marker γH2AX and HDR-mediators such as BRCA1, RPA2 and Rad51 (27). Additional evidence for HDR came from the detection of unscheduled DNA synthesis that occurred in the nucleolar caps. Interestingly, recruitment of HDR-associated repair factors and DNA synthesis occurred also in G1 suggesting that HDR may take place in rDNA repeats even in the absence of a sister chromatid, perhaps with other rDNA repeats serving as a template.

These results suggest a mechanism in human cells whereby damaged rDNA, that can not be re-joined by NHEJ factors, is recognized and repaired by the HDR-machinery at the nucleolar periphery throughout the cell cycle. It is possible that such a mechanism may serve to protect interior repeats from unscheduled recombination as it has been described in yeast (17). Relocalization of damaged repeats to the nucleolar periphery, where heterochomatinized rDNA is located, could alter the epigenetic status of the damaged locus. Heterochromatic compaction of the damaged rDNA gene may offer en advantage by limiting unscheduled recombination with other rDNA genes.

Localization of rDNA to the nucleolar periphery happens as a consequence of ATM-dependent silencing of rRNA transcription (28). However, the I-PpoI recognition site is found in the coding region of every rDNA repeat, and all the repeats are potentially cleaved upon induction of I-PpoI expression. Nucleolar caps are therefore formed in response to an unusual high number of DSBs in the rDNA and future studies will be required to address if a single or fewer DSBs will also generate sufficient ATM activation and signaling to induce nucleolar cap formation. The establishment of a cellular system with a limited number of target sequences in the ribosomal genes would allow us to study how DSBs are dealt with in the nucleoli under more physiological conditions.

In conclusion, the two above mentioned studies have yielded first insights into repair of DSBs in the nucleolar genes and thus pave the way for further investigations aimed at understanding this phenomenon in more detail and to clarify to what extent it is preserved from yeast to mammals.

**Figure 1.**
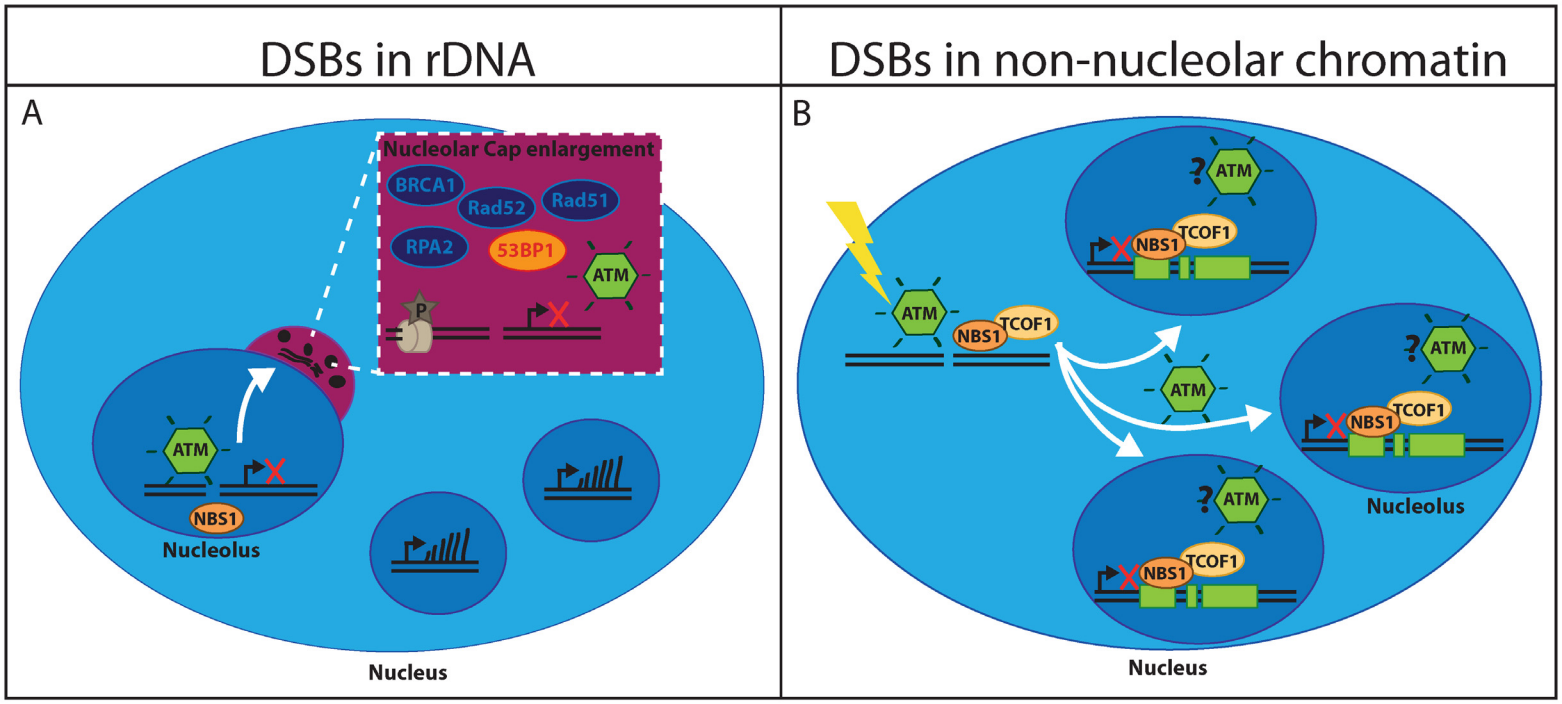
Models of rRNA silencing after DSBs. (**A**) DSBs in rDNA silence rRNA transcription locally and induce nucleolar segregation and cap formation in an ATM-dependent manner. In nucleolar caps rDNA associate with NHEJ and HDR factors in a cell cycle independent manner. (**B**) DSBs in non-nucleolar DNA induce ATM-dependent translocation of NBS1-TCOF1 into nucleoli and global silencing of rRNA transcription.

Taking these new studies in consideration, a model of the cellular response to DSBs in the nucleolus is emerging: Upon induction of DSBs in the nucleoli by either laser microirradiaiton or I-PpoI overexpression, ATM is activated locally and induces silencing of rRNA transcription within the affected nucleoli. No evidence of a response that spreads outside of the affected nucleoli was reported, which shows yet another unique feature of nucleolar chromatin as even a few DSBs induced outside of the nucleoli result in a pan-nuclear DDR (29). The DSB-induced rRNA silencing facilitate nucleolar segregation and induce the formation of nucleolar caps that may allow NHEJ and HDR factors to associate with damaged rDNA repeats in a cell cycle independent manner (see Figure 1A).

## REGULATION OF NUCLEOLAR TRANSCRIPTION IN RESPONSE TO DSBs OUTSIDE THE NUCLEOLUS

The studies summarized above described a cellular response to DSBs occurring inside the nucleoli. However, nucleolar DNA only represents 0.4% of the mammalian genome and since induction of DSBs usually occurs stochastically they affect non-nucleolar sequences with a much higher probability. The question therefore arises as to whether these DSBs would also impact on rRNA transcription and nucleolar organization. A recent paper described an *in trans* global inhibition of RNA Pol I transcription in response to DSBs introduced in nuclear chromatin outside of the nucleoli, regulated by the Treacle-NBS1 complex.

This response was associated with a rapid accumulation of the DDR factor NBS1 in the nucleoli in a manner that is spatially and temporally correlated with inhibition of rRNA transcription. Within the nucleoli, NBS1 was shown to specifically bind to actively transcribed regions of rDNA (30).

Silencing of rRNA transcription and recruitment of NBS1 are both controlled by ATM signaling as previously described for nucleolar DSB repair and rRNA silencing in response to DSB induction inside of the nucleoli. Silencing however occurred globally when induced by breaks outside the nucleoli and did not lead to nucleolar segregation and cap formation (30).

NBS1 recruitment into the nucleoli was found to be dependent on interaction with the nucleolar protein Treacle (also referred to as TCOF1). Similar findings were also reported in a study recently published by the Elledge laboratory (31). The interaction with Treacle provides the first physical link between NBS1 and the nucleolus (30,31). Treacle is a nucleolar low complexity phosphoprotein implicated in rRNA transcription and processing (32). Interestingly, the mechanism by which NBS1 is binding to Treacle shows striking similarity to its binding to MDC1, an adaptor protein that interacts directly with γH2AX in chromatin regions flanking DSBs and thus connects various DDR factors to broken chromosomes (33). Treacle and MDC1 both depend on the NBS1 FHA- and BRCT-domains and utilize a CK2-dependent phosphor-motif in Treacle and MDC1, respectively.

The studies by our group and Ciccia et al. find that accumulation of NBS1 in the nucleoli occurs in an MRE11 independent manner and thus raised the question if regulation of nucleolar transcription is conducted by the MRN-complex (composed of MRE11, RAD50 and NBS1) or if NBS1 is operating independently in this context. So far, the roles of NBS1 in the DNA damage response have predominantly been connected to its role as a subunit of the MRN-complex. In this context, NBS1 functions as an adapter module that connects the MRN-complex to DDR components such as MDC1, CtIP and ATM (34). If NBS1 has a MR-independent function in the nucleoli it may act there as an adaptor for other factors that are distinct from MRE11 and RAD50.

The precise function of the Treacle-NBS1 complex in the nucleolus remains an open issue. It is possible that rRNA transcriptional silencing in response to DNA damage is mediated by ATM-dependent phosphorylation of nucleolar factors that are implicated in rRNA transcription. In this case, Treacle-NBS1 may resume the task of transporting activated ATM from sites of DSBs into the nucleoli. This is an attractive hypothesis given that we identified several regulators of rRNA transcription that are also putative ATM targets co-immunoprecipitating with NSB1. Amongst these are the RNA polymerase I subunit RPA34 as well as TAF1C, a component of the SL1 complex. The Treacle-NBS1 complex may thus function as an adaptor for the ATM kinase to efficiently phosphorylate its nucleolar targets involved in regulation of rRNA transcription. Alternatively, phosphorylation of Treacle by ATM may itself be involved in the mechanism of DNA damage induced rRNA transcriptional silencing. In this context it is interesting to note that ATM-dependent phosphorylation of Treacle requires its direct interaction with NBS1 (30).

Another interesting observation is that the chromosomal context may influence how nuclear DSBs signal to the nucleoli. van Sluis and McStay introduce DSBs in the distal junction, a genomic sequence immediately distal to the rDNA arrays functioning to anchor ribosomal genes to perinucleolar heterochromatin. DSBs induced in this specific genomic sequence did not induce global inhibition of rRNA transcription (27). Further investigations are required to determine how inhibition of nucleolar transcription is regulated in the context of chromatin.

In summary, we propose the following model for the cellular response to DSBs in non-nucleolar chromatin: nuclear activation of ATM induces the translocation of the NBS1-Treacle complex into the nucleolus and global silencing of rRNA transcription. This response is transient and does not induce nucleolar segregation or cap formation (see Figure 1B).

## ATM: THE CENTRAL PLAYER IN rRNA SILENCING?

DSBs induced in the nucleolus or in the nucleoplasm may communicate via distinct signaling pathways but in both cases the silencing of nucleolar transcription is dependent on ATM kinase activity. It is therefore likely that identification of ATM kinase targets in the nucleoli will be the key to understand the precise mechanism of rRNA silencing in response to DNA damage. We therefore performed a gene ontology enrichment analysis of genes identified as putative ATM/ATR phopho-targets by Matsuoka *et al*. (35). Approximately 400 of the 680 genes had a cellular compartment assigned to them and amongst these 98 have been reported to localize to the nucleoli, underlining that a broad range of ATM targets are found in the nucleoli. Amongst the DNA damage-dependent ATM targets were proteins involved in rRNA transcription initiation, repression, termination and processing as well as ribosomal assembly (see Table 1). For example, the RNA Pol I subunits RPA34 and TAF1C, components of the RNA Pol I and SL1 complex, are putative ATM targets (35). As mentioned above, these putative ATM targets also co-purify with NBS1. Furthermore, the Transcription Termination Factor 1 (TTF1) associated with the T0 element upstream of the rDNA promoter and known to silence rDNA transcription by recruitment of the repressive NORC complex to rDNA (36) is modified on serine 240 (35) by ATM in response to DNA damage. Likewise the UTP14A and HEATR1, required for 28S and 18S processing respectively, are targeted by ATM at several motifs (37,38). PHF6 is another interesting putative nucleolar ATM target frequently mutated in T-cell acute lymphoblastic leukemia. PHF6 associates with UBF at the rDNA promoter region and actively suppresses rDNA transcription (39). Furthermore, NSUN5 is a conserved RNA methyltransferase regulating the structural composition of ribosomes and translation fidelity (40). Reduction of NSUN5 favors ribosomal translation of a subset of oxidative stress-responsive mRNAs (40). Regulation of NSUN5 by ATM in response to DSBs could therefore increase cellular DNA damage tolerance. Collectively, these observations highlight a number of nucleolar targets that are likely to be modified by ATM and may directly influence rRNA transcription in response to DNA damage.

**Table 1.** List of nucleolar putative ATM-targets regulating rRNA transcription

Gene name	ATM/ATR phospho site	Function in rRNA transcription
TCOF1	S1410	Stimulates transcription and processing
CD3EAP	S311	RNA Pol I subunit 34
TAF1C	S848/S858	Component of the SL1 complex
TTF1	S240	Regulates RNA Pol I transcription termination
UTP14A	S437/S445/S453	Involved in pre-rRNA processing
PHF6	T55/ S120/T358	Suppressor of rRNA synthesis
NSUN5	S432	Methylates rRNA
HEATR1	S1492	Processing of pre-rRNA

## PERSPECTIVES

Two different modes of DSB-induced rRNA silencing have been discovered: *in cis* transcriptional silencing triggered by DSBs in rDNA repeats and *in trans* silencing induced by DSBs in non-nucleolar chromatin. They are both dependent on ATM activity but it is not yet clear if they operate through common pathways and share the molecular targets.

The chain of events activated upon DSBs in rDNA is poorly understood. In this context it will be important to investigate the nature of the nucleolar DDR and the molecular mechanisms underlying rRNA silencing as well as its physiological role.

In response to non-nucleolar breaks it will be important to understand the relevance of NBS1 accumulation at rDNA. Nucleolar recruitment of active ATM by NBS1 is an attractive hypothesis due to the large number of putative ATM targets in the nucleoli. Such a role—should it be confirmed—would mirror the role of NBS1 in non-nucleolar chromatin where it was proposed to mediate the recruitment of ATM to sites of DSBs (41). Furthermore, identifying those ATM targets of relevance to rRNA silencing will provide mechanistic insight into the global regulation of rRNA transcription.

Finally, the number of stress kinases targeting rRNA transcription is growing (10). The latest addition is ATR, yet another major DDR kinase (42). Future research should clarify if rRNA silencing serves a particular purpose under specific conditions or if it represents a common cellular response to a broad variety of stresses.

The recent discoveries of the events that take place in the nucleoli in response to DNA damage have provided important clues about the connection between the DDR and ribosome biogenesis but also raised many new puzzles that future research efforts will hopefully resolve in due time.
